# Congenital malformations in Assam

**DOI:** 10.4103/0971-9261.70639

**Published:** 2010

**Authors:** Hemonta Kr. Dutta, N. C. Bhattacharyya, J. N. Sarma, Kusre Giriraj

**Affiliations:** Department of Pediatric Surgery, Assam Medical College, Dibrugarh, Assam - 786 002, India; 1 Gauhati Medical College, Guwahati, Assam - 781 006, India

**Keywords:** Congenital anomaly, congenital malformation, pediatric

## Abstract

**Aim:**

To determine the annual incidence of congenital malformations in Assam and to analyze the data.

**Materials and Methods:**

Data regarding babies born with congenital malformations in the state of Assam during the year 2006 were obtained through questionnaires and analyzed. The results were compared with similar Indian data.

**Results:**

The overall incidence of congenital malformation was 0.08%. This was considerably lower than similar published data from other states. Five hundred and eleven babies were born with congenital malformations, with 421 (82.4%) having major malformations. Males were affected more than females, 334 (65.4%) vs. 177 (34.6%). The gastrointestinal and genitourinary systems accounted for 26% and 25.8%, respectively. Malformation involving the central nervous system was more common in certain ethnic groups.

**Conclusions:**

The incidence of malformations in certain systems was at variance with the data from other states.

## INTRODUCTION

Congenital malformations are the leading cause of death in many developed countries. Many environmental factors are now recognized as potential causes of birth defects. A registry of birth defects will help in studying the malformation profile in a geographical locality and undertake etiologic studies.

## MATERIALS AND METHODS

A questionnaire was sent to all pediatricians, pediatric surgeons, general surgeons, plastic surgeons and general practitioners in Assam working in both government and private setups. All babies born during the period 1^st^ January 2006 to 31^st^ December 2006 with congenital malformations were included in this study. Data from the three medical colleges were obtained. However, only 60% of the pediatricians working outside medical colleges participated in the study. Stillborn babies with malformations could not be included in our study.

Defects that caused serious structural, cosmetic and functional disability requiring surgical or medical management were classified as major anomalies. The remaining were categorized as minor anomalies. The findings of our study were compared with the prevalence of congenital malformation in other centers.

## RESULTS

**Figure 1:**
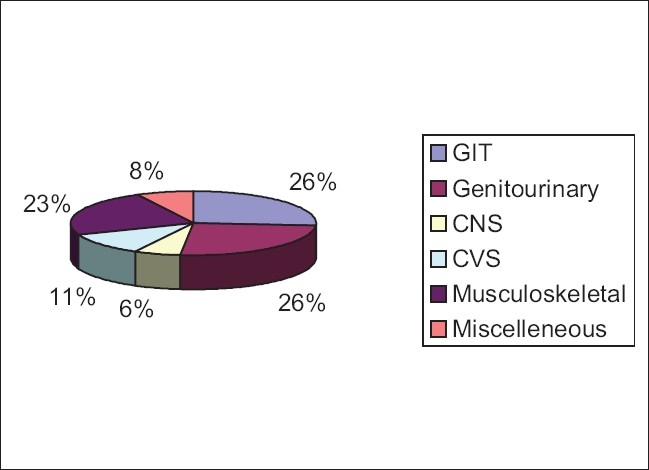
Distribution of systems involved

The total number of patients born with malformations during this period was 511. Among them, 421 (82.4%) had major anomalies and 90 (17.6%) had minor anomalies. There was a male preponderance, with 334 males (65.36%) to 177 females (34.64%). Three hundred and seventy-three patients (73.9%) were from the Assamese community, followed by 42 from the Tea tribes (8.22%). Four hundred and eighteen patients (81.8%) were Hindu, 66 were (12.9%) Muslims and 27 (5.28%) were from other religious sects. Malformations involving the gastrointestinal tract (26%) and genitourinary tract (25.8%) were the most common anomalies [Figure 1]. Facial defects were the next most common anomaly (17.4%). A significant number had malformations involving the central nervous system.

Some rare anomalies like anterior encephalocele were also noted in a few. Mortality in live births due to congenital malformation was 0.008%.

The total population of Assam is 28,665,000, with a birth rate of 24 per 1,000. In the present study, the overall incidence of congenital malformation in the live born babies is 0.08%. We have observed that the prevalence of birth defects in Assam is significantly lower as compared with few other places in India.

## DISCUSSION

The overall incidence of congenital malformations in India ranges from 0.3% to 3.6%.[1–3] It is higher in centers where autopsy is carried out as a routine.[4] The prevalence of birth defects was found to be significantly lower in Assam than in compared groups, with the overall incidence of congenital malformation in the live born babies being only 0.08%. One reason for this difference is probably the inclusion of only those who sought medical care from physicians.

Stoltenberg *et al.* reported that the risk of birth defects is practically equal for all children with nonconsanguineous parents, independent of ethnic origin.[5] They also observed that the risk of birth defects was higher in populations with a higher frequency of consanguineous marriages. The incidence of consanguineous marriages in Assam is just 1.4%[6] compared to 55% in Pondicherry and 26.4% in Maharashtra.[7] The significant difference in the prevalence of birth defects in Assam vs. Maharashtra and Pondicherry may also be due the differences in the methodology and the year of study. In a study performed at Wardha, India, the incidence of congenital malformation was found to be 2.72%.[8] An increase in frequency was seen in advanced maternal age and in primi and fourth gravida mothers. Outside India, the incidence varied from 0.9% in Northampton Shire[9] to 3.4% in Michigan[10] and 5.5% in Afghanistan.[11]

**Figure 2:**
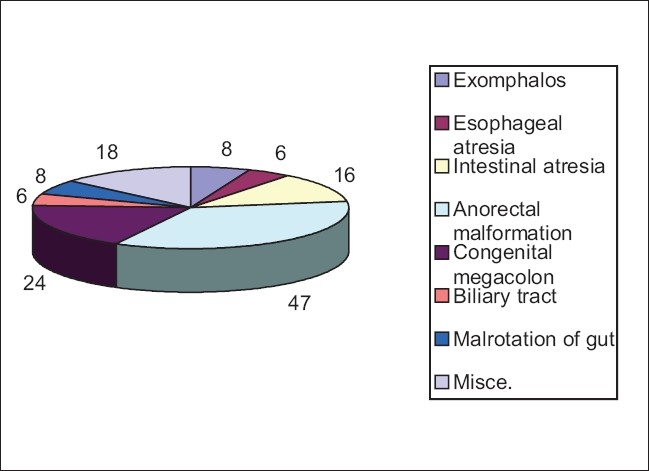
Distribution of GIT malformations

**Figure 3:**
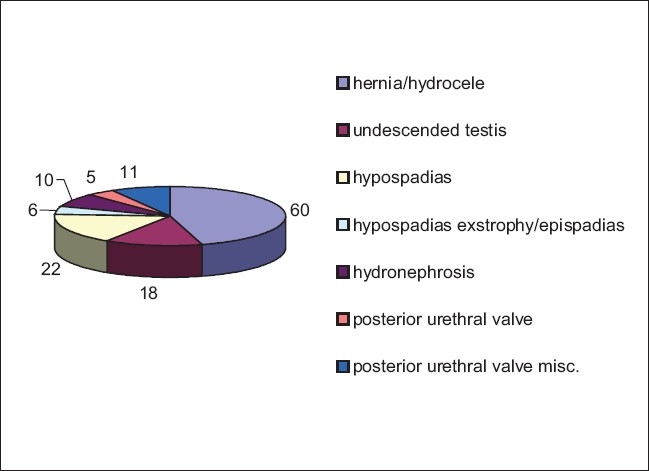
Distribution of GUT malformations

The most common system involved in our study was the gastrointestinal system [Figure 2], followed closely by the genitourinary tract [Figure 3]. The musculoskeletal system[12] and the central nervous system[13] were the most commonly involved systems (Davanagere, Karnataka)[13,14] in other studies.

One interesting observation in this study has been the report of three cases of anterior encephalocele, which is rare in other parts of the country, and still rarer in the West. All the three were from the ethnic Tea garden community, in whom other central nervous system (CNS) malformations are also frequently seen. In our study, only 29 (5.6%) patients had CNS malformation. The reason for this lower incidence may be the prevalence of nonconsanguineous marriages in Assam. Anterior encephalocele, although rare in India, is very common in the neighboring Burma and Thailand.[15] Because of the geographical proximity of Assam with these two countries, it is conjectured that the etiological factor may be similar.

The present study is a preliminary one to obtain firsthand information about the magnitude of the problem of congenital malformations in our state. The Birmingham Malformation Register data showed that data for a single year was not considered complete until ascertainment had continued for 6 years after birth, by which time the malformation rate had risen from 19.1 per thousand to 26.7 per thousand.[16] The study also emphasized the fact that environmental teratogenic effects must be involved in the etiology of many malformations and that, among the viruses, several strains of Coxsackie A and B and Echoviruses may be involved in a chronic or recurring manner. The incidence of congenital malformation is also six-times higher among still births. In the present study, we could not record the still births as data were collected only from patients seeking medical care.

We propose to follow-up with another study that will include the following: (a) record of the still births and associated malformation if any and (b) maternal history – maternal age, consanguinity, illness during pregnancy, past abortion, presence of poly or oligohydramnios and history of any medication during pregnancy.
